# Labyrinthine Fistulae in Squamosal Type of Chronic Otitis Media: Therapeutic Outcome 

**Published:** 2019-05

**Authors:** Priyanka Misale, Anjali Lepcha, Ramanathan Chandrasekharan, Manusrut Manusrut

**Affiliations:** 1 *Department of Otorhinolaryngology, Unit-4(Audio Vestibular Diseases/Neurotology),Christian Medical College, Vellore- 632004, India.*; 2 *Department of Otorhinolaryngology, Badr Al Samaa Hospital Salalah, Sultanate of Oman (Previously-Dept of ENT-4, Christian Medical College, Vellore, India).*; 3 *Department of Otorhinolaryngology, District Hospital, Hyderabad, India (Previously-Dept of ENT-4, Christian Medical College, Vellore, India.)*

**Keywords:** Cholesteatoma, Hearing Loss, Labyrinth diseases, Middle Ear, Vertigo

## Abstract

**Introduction:**

Labyrinthine fistulae (LF) are the common complications of chronic otitis media (COM) of squamosal variety. The final therapeutic outcome of this condition is to preserve the cochlear and vestibular functions. Herein, we present the data of the cases managed at our institute with respect to their presenting complaints, adopted therapeutic approaches and outcomes.

**Materials and Methods:**

A retrospective chart review was conducted on all cases with COM squamosal type in adult patients. A total of 275 patients were reviewed, out of whom 30 cases had LF. The results were mainly studied with respect to the postoperative improvement of hearing and vertigo.

**Results:**

The incidence rate of LF in the present study was obtained at 10.9%. Only 50% of the cases had the symptoms of vertigo. Furthermore, positive fistula test was elicited in 3.3% of the cases. All cases undergoing preoperative imaging were diagnosed successfully. In addition, 42.85% of the cases had profound hearing loss preoperatively, which sustained after the operation. However, 47.61% of the cases showed an improvement of at least ≥ 10 dB in the air-bone gap. Out of the 15 LF cases with vertigo as the main complaint, only 11 cases referred for follow-up. In this regard, 63.63% of the cases had no postoperative vertigo symptoms.

**Conclusion:**

Patients with LF may not have complaints of vertigo and a positive fistula sign upon admission. Pre-operative imaging facilitates the diagnosis of this condition. The removal of the matrix under constant irrigation, followed by repair with bone wax and/or autologous tissue, is sufficient to preserve the cochlear and vestibular symptoms postoperatively.

## Introduction

Labyrinthine fistula (LF) is a common complication of chronic otitis media (COM) squamosal type with an incidence rate varying from 4% to 12.7% (1-10). About 50-80% of LF cases may present with vertigo (7-14), along with the other features of COM-like ear discharge and hearing loss. According to the literature, a positive fistula sign presents in around 9-55% of LF cases (7-16). The goal of LF treatment is to preserve the cochlear and vestibular functions while eradicating the disease. Some studies have reported decreased level of hearing after operation (10-14,17), which can be profound depending on the extent of fistula and the associated disease.

Various methods have been described for the management of these fistulae in the medical literature. A group of researchers support the removal of matrix from the fistula site (9,16-19), followed by its repair, while the other group advocates the exteriorization of the cavity and leaving the fistula site covered with matrix. Yet, another group supports removal based on the extent of the disease (6,8,10,20). Hearing outcomes following surgery for LF have been reported to be poor. With this background in mind, the present study was conducted to investigate patients with COM squamosal type with LF who were surgically managed at our institute. 

## Materials and Methods

A retrospective chart review was conducted on the adults suffering from COM squamosal type between January 2014 and December 2016. A total of 275 cases were identified, 30 cases of whom had LF that was confirmed intraoperatively. The inclusion criteria were: 1) LF secondary to erosion by cholesteatoma, 2) diagnosis of LF types 2, 3, and 4 according to the Ramsay and Palva classification (21), and 3) a minimum postoperative follow-up of 3 months. Both primary and revision cases were included in the study. The exclusion criteria were: 1) LF type 1 (according to the Ramsay and Palva classification), 2) no follow-up or a follow-up period shorter than 3 months, and 3) LF due to causes other than cholesteatoma. 

The preoperative clinical signs and symptoms, imaging findings, and hearing levels were noted for all participants. Details of intra-operative findings and repair were gathered, along with the postoperative outcomes in relation to changes in hearing level and vertigo. In addition, the last pre- and postoperative audiograms were analyzed to determine the total improvement in air bone gap (ABG) following the LF repair. This study was approved by the institutional review board (IRB min No. 10723 [retro] dated 21.06.2017).

**Statistical analysis**

The demographic data, surgical records, pre- and postoperative audiological data, and follow-up records were obtained from the computerized database maintained at our institution. The data were analyzed in MS Excel. The baseline variables were presented using descriptive statistics. Four frequency averages (i.e., 0.5, 1, 2 and 4 kHz) were obtained and used to calculate the pre- and postoperative ABG, air conduction (AC), and bone conduction (BC). The postoperative hearing outcomes were categorized into three groups as follows: Group A: those who had a definite improvement in hearing (≥10 dB closure of ABG) or those with unchanged hearing (no change in ABG, AC, or BC across four frequencies); Group B: those with the deterioration of hearing as demonstrated by the worsening of BC across four frequencies or the elevation of the ABG values by ≥ 10 dB postoperatively; Group C: those who had a dead ear postoperatively.

**Surgical procedure**

**Fig 1:**
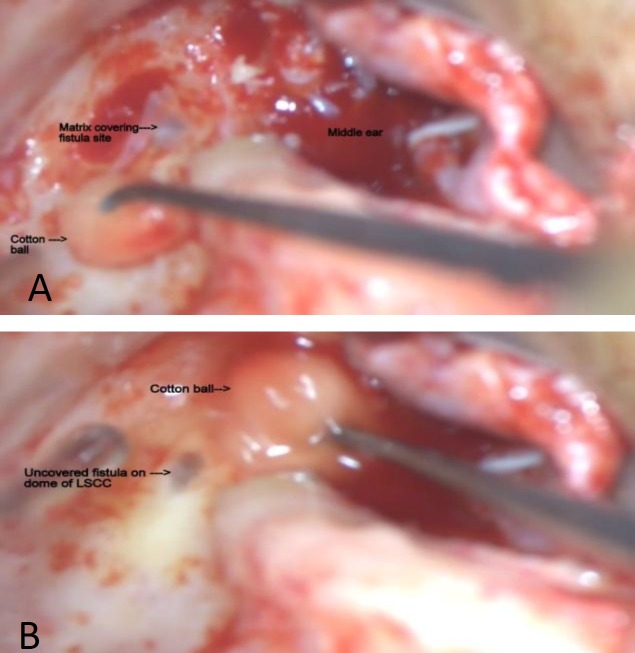
A: Cholesteatoma matrix covering the fistula site on the lateral semicircular canal, B: Matrix clearance off the fistula site under continuous irrigation

All cases, though operated by different surgeons, were subjected to the same protocol. In this regard, the surgery was started with the clearance of the cholesteatoma and eradication of disease. The site of suspected fistula was managed at the end of the surgery. To this end, the matrix was gently dissected off the fistula site under copious irrigation; thereafter, the site was immediately sealed off using either bone wax followed by temporalis fascia or a sandwich of the temporalis fascia, conchal cartilage, and temporalis fascia (Figs 1A and 1B). All cases were followed up for a minimum period of 3 months after the operation. The obtained results were studied with respect to postoperative improvement in hearing and vertigo symptoms, as well as the eradication of the disease.

## Results

Within the study period, a total of 275 patients with COM were identified, 30 (10.9%) cases of whom had LF. Out of the 30 patients with LF, 16 (53.3%) and 14 (46.6%) cases had fistulae in their left and right ears, respectively. The LF patients consisted of 25 (83.3%) males and 5 (16.6%) females with the mean age of 34.03(SD 14.3) years (age range: 18-72 years). Out of the 30 patients, 26 (86.6%) and 4 (13.3%) subjects underwent primary and revision surgeries respectively.

With respect to the clinical symptoms, 29 (96.6%), 27 (90%), and 15 (50%) patients had hearing loss, ear discharge and vertigo as their main complaints respectively. Furthermore, the symptoms of facial palsy, tinnitus, and aural pain were present in 1 (3.3%), 2(6.6%) and 5(16.6%) patients respectively (Table.1). 

**Table 1:** Preoperative symptoms, tests used for preoperative diagnosis, and sites of fistulae in previous studies and our research

**STUDY**	**N**	**Pre-operative symptoms**					**Pre-operative diagnosis**		**Sites of fistulae**				
		**Ear discharge**	**Vertigo**	**Hearing loss**	**Tinnitus**	**Facial nerve involvement**	**Fistula test**	**Pre-op** **CT scanning**	**LSCC (%)**	**SSCC (%)**	**PSCC (%)**	**Multiple (%)**	**Promontory (%)**
Sheehy (1979)	97	-	65%	-	-	50%	31%	-	86.5	0	0	14.43	3.09
Palva (1989)	84	-	-	-	-	-	25%	-	90.47	0	2.3	0	7.14
Dornhoffer (1995)	37	79%	62%	-	11%	-	32%	-	86.48	41.8	0	2.7	5.4
Soda Merhy(2000)	27	93%	78%	100%	44%	11%	55.5%	91.6	100	7.40	0	11.1	3.7
Quaranta (2009)	46	-	43%	-	-	6.5%							
Kobayashi (1989)	5		100%				33.3%	100%	100	0	0	0	0
Glasscock (1990)	4		-				-	75%	100	25	0	25	0
Gersdorff (2000)	64		57.4%				9.25%	48%	60.9	18.75	4.6	17.1	9.3
Herzog (1996)	17		52.9%				41.17%	37.5%	94.1	0	0	0	5.88
Parisier (1991)	41		75%				22.5%	53.3%	82.9	14.6	0	14.63	0
Kvestad (2001)	20	90%	50%	96.6%	16.6%	3.3%	20%	55%	90	10	5	15	10
Manolides (2000)	23					73.9%	-		91.3	8.6	0	8.6	8.6
Sanna (1988)	158					-	-		91.1	0	0	10.75	8.8
Our study (2017)	30	90%	50%	96.6%	6.6%	3.3%	3.3%	100%	86.6	0	6.6	6.6	0

LSCC: lateral semicircular canal, SSCC: superior semicircular canal, PSCC: posterior semicircular canal

Positive fistula test was elicited in only one of the 30 patients, and 9 (30%) patients had profound deafness prior to the surgery based on the pure tone audiometry. The average pure tone threshold before the operation was 57.91 dB. In the ear examination, posterosuperior retraction pocket with cholesteatoma was the most common finding (n=13,43.3%), followed by polyp in the external auditory canal (n=5, 16.6%) and attic perforation (n=4, 13.3%). In addition, some patients also presented with granulation tissue in the external auditory canal with canal narrowing (6.6%), previous modified radical mastoidectomy cavities (10%), canal cholesteatoma (3.3%), and automastoidectomy cavities (6.6%). 

Out of the 30 patients with LF, only 26 cases had undergone high-resolution computed tomography (HRCT) of the temporal bone. All fistulae of type 2 and above were detected by imaging. All patients who had undergone preoperative scanning and were diagnosed with LF were confirmed to have LF intraoperatively. The lateral semicircular canal dome was affected in 26 (86.6%) patients. Two cases (6.6%) were detected with multiple fistulae affecting the lateral and posterior semicircular canal in one patient and the lateral and superior semicircular canal in the other. Furthermore, the posterior canal was solely affected in 2 (6.6%) patients.

Out of the 29 patients with hearing loss as one of their main complaints, only 21 subjects came for follow-up after the surgery. Since the hospital under investigation is a referral center, it welcomes many patients from distances as far as 2,000 km away and some even from other countries. The follow-up of the patients ranged from 3 to 30 months with a mean duration of 5.2 months. 

**Fig 2:**
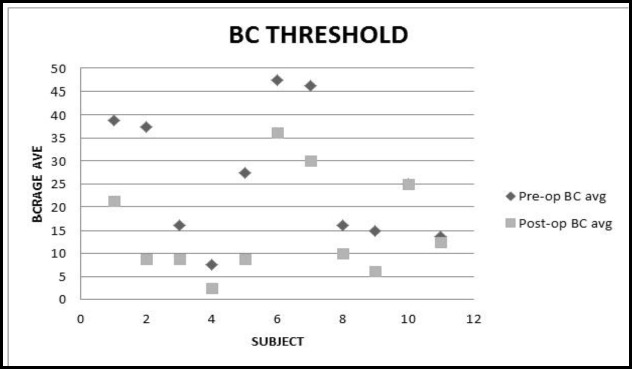
Scatter plot showing preoperative and postoperative bone conduction threshold

Based on the results, 9 (42.85%) patients had preoperative and postoperative profound hearing loss. A total of 10 (47.61%) subjects had an improvement in the ABG of ≥ 10 dB; however, the hearing remained unchanged in 2 (9.52%) patients (Fig.2, Table.2). There was no LF case with the deterioration of ABG or BC threshold after the surgery.

**Table 2:** Hearing outcomes following the surgery in previous studies and our research

**Study**	**Dead ear pre-op**	**Hearing improved/unchanged**	**Hearing decreased**	**Dead ear post-op**
Ostri (1989)	15% (3/20)	76.4% (13/17)	17.64% (3/17)	5.8% (1/17)
Kobayashi (1989)	0% (0/5)	100% (5/5)	0% (0/5)	0% (0/5)
Dornhoffer (1995)	11% (4/37)	78.7% (26/33)	0% (0/33)	21.2% (7/33)
Pulec (1996)	34% (32/95)	100% (63/63)	0% (0/63)	0% (0/63)
Herzog (1996)	0% (0/17)	94% (16/17)	6% (1/17)	0% (0/17)
Soda Merhy (2000)	15% (4/27)	87% (20/22)	13% (3/23)	0% (0/23)
Our study (2017)	43% (9/21)	100% (12/12)	0% (0/12)	0% (0/12)

Out of the 15 LF cases with giddiness as the main complaint, only 11 patients referred for follow-up. Among these 11 patients, 7 (63.63%) patients had no postoperative symptoms of giddiness, while 4 (36.36%) had persistent vertigo postoperatively, 2 of whom were found to have associated vertiginous migraine and were treated appropriately. 

Regarding the other two mentioned patients, one was a 72-year-old elderly gentleman who had other associated problems of cataract, hypertension, diabetes mellitus and had suffered stroke on two occasions leading to hemiparesis. Therefore, it was difficult to accurately pinpoint the contribution of vestibular pathology to his sense of imbalance and dizziness. The other patient reported a change in the character of dizziness from his initial sense of surrounding rotatory vertigo to a more generalized sense of imbalance. He was put on vestibular rehabilitation exercises. All patients who came for follow-up had a dry ear at their first follow-up session (around 3 months).

## Discussion

The incidence rate of LF in patients with COM squamosal type was obtained at 10.9% in the present study which is similar to the rates reported in previous studies (1,7,8). Compared to other studies (6,10), in our study, LF had a higher incidence in males (83%) than in females (17%). Our results revealed no significant difference between the involvement of the right (46.6%) or left ear (53.3%), which is in line with the results obtained by Dornhoffer et al.(9). 

In the current study, primary surgeries were carried out in 26 (86.3%) cases, and revision surgeries were performed in 4 (13.3%) cases. We had a lower rate of LF detection in revision surgeries when compared to that reported by Soda Merhy et al. (10) who performed primary and revision surgeries in 59% and 41% of patients, respectively. This difference may be due to the longer study period (144 months) and follow-up (13 years) in the mentioned research. 

In our study, hearing loss and ear discharge were observed in 96.6% and 90% of the cases respectively, which is in close agreement with the rates obtained in a study conducted by Soda-Merhy et al. (10). Vertigo as a complaint was present in 50% of the cases when compared to the other studies which reported the presence of vertigo in 43%, 65%, 62% and 78% of their subjects (6,7,9,10). Tinnitus was detected in 6.6% of the cases; however, this rate was obtained at 11% and 44% by Dornhoffer et al. and Soda Merhy et al. (9,10), respectively. Additionally, facial nerve involvement was observed in 3.3% of the cases, while Soda Merhy et al. and Sheehy et al. reported it in 11% and 50% of the patients respectively (7,10). 

Only 3.3% of the cases elicited a positive fistula sign as compared with the relatively higher values noted by Soda Merhy et al., (10) (55%), Dornhoffer et al., (9) (32%), Sheehy et al., (7) (31%), and Palva et al. (16) (25%). As our study was a retrospective chart review, we had no control over the data obtained. Probably, the failure of mentioning the outcome of fistula test in the case sheets resulted in the lower rate of positive fistula sign. In such cases where there was no remark about the outcome, the result was considered as a negative fistula sign.

Based on the symptoms of vertigo, LF was suspected in 15 (50%) patients. Sheehy et al., Dornhoffer et al., Soda Merhy et al., Kobayashi et al., and Gersdorff et al., observed vertigo as a complaint in 65%, 62%, 78%, 100%, and 57.4% of the patients respectively (7,9,10,13,15). All the patients who underwent preoperative imaging were diagnosed with LF of stages type 2 and above. In the same vein, this detection value was reported as 100% in a study performed by Kobayashi et al. (15). However, in the other studies, the detection of LF by imaging was less than 100% (8,10,11,13,14). 

This difference in the detection rate of LF by preoperative imaging among various studies could be ascribed to the use of different fistula staging and also our inclusion criteria which only included those cases with fistulae type 2 and above. The applied protocol of HRCT and the thickness of the slices obtained on CT scanning could also be contributing factors in the detection of LF preoperatively on scan. 

Similar to the other reports, in the present study, lateral semicircular canal (86.6%) was the most frequently involved region. Based on our findings, the posterior semicircular canal was affected in 6.6% of the cases; however, in the study by Palva et al. (16), this rate was reported as 2.2%. In the current research, 6.6% of the patients had fistulae involving multiple canals. This rate was obtained at 14.4%, 10.75%, and 8.6% in the studies performed by Sheehy et al., Sanna et al., and Manolides(2,5,7) respectively. There was no promontory/cochlear fistula detection in our series unlike those reported in other studies (2,5,7,9,16).

For comparative purposes, the postoperative hearing outcomes were categorized into three groups of A, B, and C. Group A involved those who had a definite improvement of ≥ 10 dB or those with unchanged hearing. Group B consisted of those with decreased hearing. Finally, group C comprised those with a dead ear postoperatively. In the present study, all the cases belonged to group A, which is similar to the studies by Pulec (100%) and Kobayashi et al. (100%) (3,15). Accordingly, we did not encounter any patients with decreased hearing or dead ears postoperatively unlike that observed by some researchers (9-11,17). In our study, 63% of the patients were relieved of their vertigo symptoms as compared to the rates reported by Soda Merhy et al. and Chen et al., who observed an improvement in 96% and 100% of the patients, respectively (10,22).

## Conclusion

The LF may not always be accompanied by the symptoms of vertigo and a positive fistula sign. Therefore, a high index of suspicion is required in all cases of COM squamosal type. The HRCT of the temporal bone is highly sensitive in diagnosing LF preoperatively; accordingly, it is recommended to perform this imaging modality for all patients with squamosal type of COM. Dissection of the cholesteatoma matrix under copious irrigation facilitates the hydrodissection of the matrix from the fistula site. This helps to prevent the incidence of dead ear during surgery by accidental perilymph suction and damage to the membranous labyrinth thereby preserving the inner ear function. Careful surgical technique appears to result in good postoperative outcomes with respect to hearing and vertigo symptoms.
